# Renal injury, nephrolithiasis and *Nigella sativa*: A mini review

**Published:** 2016

**Authors:** Parichehr Hayatdavoudi, Abolfazl Khajavi Rad, Ziba Rajaei, Mousa AL-Reza Hadjzadeh

**Affiliations:** 1 *Neurocognitive research center & department of physiology, Faculty of Medicine, Mashhad University of Medical Sciences, Mashhad, Iran*; 2 *Neurogenic inflammation research center & department of physiology, Faculty of Medicine, Mashhad University of Medical Sciences, Mashhad, Iran*; 3 *Department of physiology, School of Medicine, Isfahan University of Medical Sciences, Isfahan, Iran*

**Keywords:** *Nigella sativa L*, *Thymoquinone*, *Herbal melanin*, *Nephrolithiasis*, *Rena failure*

## Abstract

**Objective:**

The incidence and prevalence of kidney stone is increasing worldwide. After the first recurrence the risk of subsequent relapses is higher and the time period between relapses is shortened. Urinary stones can be severely painful and make a huge economic burden. The stone disease may increase the vulnerability of patients to other diseases such as renal failure. Medicinal herbs are rich sources of antioxidants which are increasingly consumed globally for their safety, efficacy and low price. *Nigella sativa* is a spice plant that is widely used for prevention and treatment of many ailments in Muslim countries and worldwide. This review aims at investigation of the effects of *Nigella sativa* on renal injury and stone formation.

**Materials and Methods:**

The scientific resources including PubMed, Scopus, and Google scholar were searched using key words such as: nephrolithiasis, urolithiasis, kidney/renal stone, renal injury, renal failure, urinary retention and black seed, black cumin, *Nigella sativa* and thymoquinone.

**Results:**

*N. sativa* and its main component, thymoquinone showed positive effects in prevention or curing kidney stones and renal failure through various mechanism such as antioxidative, anti-inflammatory, anti-eicosanoid and immunomodulatory effects. The putative candidate in many cases has been claimed to be thymoquinone but it seems that at least in part, particularly in kidney stones, the herbal melanin plays a role which requires further investigation to prove.

**Conclusion:**

*N. sativa* and its components are beneficial in prevention and curing of renal diseases including nephrolithiasis and renal damages.

## Introduction

Kidney stones are formed when the balance of crystal inhibitors and accelerators is interrupted in the urine (Deepika et al., 2013 ▶); however, the renal morphology also plays an important role in promotion of crystal precipitation (Grases et al., 2006 ▶). The incidence and prevalence of kidney stones progressively increase worldwide (Romero et al., 2010 ▶), its prevalence is about 5-10% and the recurrence rate is about 50% (Chou et al., 2011 ▶). After the first relapse, the risk of subsequent relapses is higher and the time period between relapses is reduced (Moe, 2006 ▶). Kidney stone is three times more expected in men than women (Mckenzie and Hall, 2013 ▶) except in the sixth decade of life (Moe, 2006 ▶). The most frequent form of nephroliths is calcium oxalate and calcium phosphate (CaOx) stones (Coe et al., 2005 ▶) with more than 80% occurrence rate in the population (Moe, 2006 ▶). Types and incidence of kidney stones were shown in Table 1(Coe et al., 2005 ▶; Sayer et al., 2010 ▶).

**Table 1:** Types of kidney stones in USA

**Types and incidence of kidney stones**	
**Type **	**Incidence**
Calcium oxalate	60%
Calcium phosphate	20%
Uric acid	9-10%
Struvite	8-10%
Cystine	0.5-1.5%
Other : xanthine stones, drug induced stones (indinavir, triamterene), 2,8 dihydroxyadenin (2,8-DHA) stones	0.5%

Nephrolithiasis is a complicated multifactorial process which is concomitant with some inflammatory responses but, it is unclear that these events are primary or secondary to nephrolithiasis (Merchant et al., 2008 ▶). In some cases, patients with renal stones suffer from cardiovascular diseases, chronic kidney disease and cancer, also it is demonstrated that inflammation and oxidative stress play a role in kidney stone formation (Tsao et al., 2007 ▶). Kidney stones can be severely painful (Alok et al., 2013 ▶) and their treatment has a huge economic burden (Nouvenne et al., 2008 ▶). The prevalence of kidney stones has an ancient history and they are found all over of urinary tract (Alok et al., 2013 ▶). 

There are different therapeutic methods for kidney stones such as the extra corporeal shock wave lithotripsy (ESWL), percutaneous nephrolithatomy (PCNL) and open surgery but these methods show side effects, for example ESWL procedure has occasional complications such as infection, hematoma and adjacent organ damage (Mckenzie and Hall, 2013 ▶); although, Irani et al. (2005) ▶ reported that outpatient ESWL is nonhazardous and acceptable even in complicated cases such as ureteral stones, single kidney patients and children (Irani et al., 2005 ▶). Moreover, it should be kept in mind that a lithotripter is not capable of prevention of stone formation; therefore, drug therapy is used in nephrolithiatic patients to prevent stone formation. The most frequently used drugs for nephrolithiasis are thiazides, allopurinol, and potassium citrate (Heilberg, 2000) in addition to, etidronate disodium and zonisamide (Alok et al., 2013 ▶). 

Kidney stone increases the vulnerability of patients to renal failure. The risk factors such as anatomical malformations lead to obstructive nephropathy, infection and inflammation. Moreover, metabolic disorders including chronic urate nephropathy or diabetes mellitus, hypertension, environmental and dietary factors predispose the nephrolithiatic patients to renal function disturbance (Chou et al., 2011 ▶). Unfortunately, the clinical consequences of stone disease are not fully identified (Chou et al., 2011 ▶). All urinary calculi can lead to infection secondary to urinary stasis and obstruction. Furthermore, chronic inflammation causes the ipsilateral kidney function disturbance, xantho-granulomatous pyelonephritis and squamous cell carcinoma especially in patients with a history of staghorn stones or bladder stones (Mckenzie & Hall, 2013 ▶). The incidence of a unilateral ureterovesicular junction obstruction due to stone disease is claimed at 20% in literature; however, bilateral ureteral stones are not common, although acute renal failure following bilateral obstructing renal calculi has been reported (Stone et al., 2010 ▶). In obese patients, it has been reported that bariatric surgery leads to advanced renal failure as a consequence of secondary hyperoxaluria, therefore, such surgery is followed by oxalate nephropathy and nephrolithiasis (Karaolanis et al., 2014 ▶). Also, Crohn’s disease predisposes the patients to kidney stones because of secondary enteric hyperoxaluria (Karaolanis et al., 2014 ▶). Therefore, treating the underlying diseases may lead to the prevention of stone occurrence and treatment of kidney stones may also prevent consequential nephropathies.

Medicinal herbs are rich sources of antioxidants and are used in both industrialized and developing countries. People prefer them because they believe herbal drugs have not any significant side effects. *Nigella sativa* is a spice plant that is widely used for prevention and treatment of many ailments in Muslim countries. It is from *Ranunculaceae* family and is known as black seed, black cumin, fennel flower and kalonji (Kanter et al., 2005 ▶). This review focused on the effects of *N. sativa* on renal injury and stone formation.

***Nigella sativa*** **Linn**

*N. sativa* is an annual plant from Ranunculaceae family. Analysis of *N. sativa* seeds has revealed that it contains following ingredients: alkaloids, saponin, proteins, 36-38% fixed oils and 0.4- 2.5% essential oil.

High performance liquid chromatography shows that the main active constituents in essential oil are thymoquinone, dithymoquinone, thymohydroquinone and thymol (Ragheb et al., 2009 ▶) although, thymoquinone is also present in the fixed oil (Ali and Blunden, 2003 ▶).

## Materials and Methods

**Search strategy**

 The scientific resources including PubMed, Scopus, and Google scholar were searched using key words such as: nephrolithiasis, urolithiasis, kidney / renal stone, renal injury, renal failure, urinary retention and black seed, black cumin, *Nigella sativa* and thymoquinone. Table 2 represents a summary of associated articles. 

**Table 2:** Studies about renal damage and kidney stone and* N. sativa* *L*. treatment

**Model**	**species**	**Type of extract**	**Reference/ year**
**Kidney stone**	Rat	Ethanolic	( Hadjzadeh et al., 2007)
**Kidney stone**	Rat	Ethyl acetate phase remnant fraction	( Khajavi Rad et al., 2008)
**Kidney stone**	Rat	Thymoquinone	( Hadjzadeh et al., 2008)
**Kidney stone**	Rat	N-butanol fraction of 50% aqueous ethanolic	( Hadjzadeh et al., 2011).
**Renal failure**	Rat	Thymoquinone	(Sayed-Ahmed & Nagi, 2007)
**Hypertension & renal damage**	Rat	Thymoquinone	(Khattab & Nagi, 2007)
**Ischaemia/reperfusion renal injury**	Rat	Nigella sativa oil	(Bayrak et al., 2008)
**Ischaemia/reperfusion renal injury**	Rat	Thymoquinone	(Awad et al., 2011)
**Cisplatin- induced Kidney injury**	Rat	Thymoquinone	(Ulu et al., 2012)
**Bromobenzene hepato-renal toxicity**	Rat	Nigella sativa seed oil	(Hamed et al., 2013)
**Acetaminophen-induced renal impairment**	Rat	Nigella sativa oil	(Ahmed, 2013)
**Ischaemia/reperfusion kidney damage**	Rat	Hydroalcoholic extract	(Havakhah et al., 2014)
**------**	Kidney tubule epithelial cells	Thymoquinone	(Vance et al., 2008)

**Renal injury models**

*Acetaminophen-induced renal injury*

There are a few reports about the effects of *N. sativa* or thymoquinone on renal function. Ahmed (2013) ▶ has reported that *N. sativa* oil (NSO) has renal protective effects against acetaminophen-induced renal injury in rats. According to his study NSO had beneficial effects both at low dose (2ml/kg) in chronic treatment and high dose (4 ml/kg) in acute experiments. NSO increased viability of animals, decreased arterial blood pressure and modified urinary and plasma biomarker alterations. Furthermore, pretreatment with NSO prevented the kidney from structural changes.

*Bromobenzene-induced hepatorenal injury*

The black cumin seed oil efficiently inhibited collagen deposition and the severity of fibrosis in a bromobenzene-induced hepato-renal injury model. Also, it stabilized the membrane permeability, reduced the liver enzyme leakage in the circulation and improved renal function parameters (Hamed and Ali, 2013 ▶).

**NG-nitro-L-arginine methyl ester (L-NAME) induced hypertension and renal injury**

Following L-NAME- induced hypertension and consequential renal damage model, oral thymoquinone supplementation improved renal GSH (glutathione) level, creatinin and systolic blood pressure after third and fourth weeks of treatment (Khattab and Nagi, 2007 ▶).

**Cisplatin-induced renal injury**

Ulu et al. (2012) using a cisplatin-induced renal damage model, showed that thymoquinone supplementation protected the kidneys with increasing of organic anion (OAT1 and OAT3) and cation (OCT1 and OCT2) transporters and reducing efflux transporters, (i.e, multidrug resistance-associated proteins (MRP2 and MRP4)). 

Furthermore, thymoquinone decreased the oxidative and lipid peroxidation markers, MDA and 8-isoprostane levels in renal tissues and reduced levels of serum creatinin and urea concentration.

**Ischemia reperfusion-induced renal injury**

Study of Havakhah et al. (2014) ▶ showed *N. sativa* hydroalcoholic extract (NSE), 150 and 300 mg/kg reduced kidney oxidative stress and MDA level with increment of thiol groups in two protocols of preventive and treatment groups. Furthermore, NSE preserved renal morphology and DNA structure post-treatment. However, pretreatment with NSE did not affect DNA damage significantly. 

In another study, Bayrak et al. (2008) ▶ showed that *N. sativa* oil (NSO) improved serum BUN and creatinin levels, in ischemia/reperfusion injury (I/R) model, also enhanced serum superoxide dismutase (SOD) and glutathione peroxidase (GSH-Px) activities and increased tissue catalase (CAT), GSH-Px and SOD levels. Malondialdehyde content of serum and tissues decreased by NSO compared to I/R group. NSO treatment reduced total oxidant capacity (TOC) and increased total antioxidant capacity (TAC), reduced the nitric oxide (NO) and protein carbonyl content and normalized the histopathological scores compared to I/R groups. These beneficial effects were attributed to free radical scavenging effects of NSO.

**Gentamicin-induced acute renal toxicity**

According to Ahmed and Nagi (2007) ▶ study, oral thymoquinone (TQ) supplementation not only prevented ATP reduction but also increased it as well as ATP: ADP ratio. Increment of this ratio is essential for proper mitochondrial functioning. Based on their results, substrate utilization and / or oxidative phosphorylation may be increased by TQ. TQ enhanced energy production and mitochondrial function, and completely restored the GSH, GPx and CAT levels,that were decreased by gentamicin. Also, TQ reduced total NO_x _(index of NO production) to control values. NO is important to modify acute renal failure due to gentamicin. 

**Nephrolithiasis**

There are a few reports about *N. sativa* and / or thymoquinone and renal stones. Hadjzadeh et al. (2007) ▶ have reported that oral intake of ethanolic extract of *N. sativa* for 30 days not only has insignificantly prevented the formation of calcium oxalate (CaOx) deposits in prevention groups but also reduced CaOx depositions in treated groups by 57%. The extract did not cause any effect on kidney weight, because the treatment time was short. The urine oxalate level also significantly decreased in prevention group compared to ethylene glycol group and no significant difference was observed between treatment and control groups. In another set of studies, the preventive action of N-butanol and N-butanol phase remnant fractions against renal calculi was more powerful than 50% ethanolic extract (Hadjzadeh et al., 2011 ▶). They have also tested thymoquinone, the major component of *N. sativa*, and indicated that intraperitoneal injection of thymoquinone for 28 days had preventive and disruptive effects on the CaOx deposits (Hadjzadeh et al., 2008 ▶). The ethyl acetate phase remnant fraction also showed significant preventive effects against renal calculi while, the ethyl acetate fraction by itself did not show any effect (Khajavi rad et al., 2008 ▶).

**Kidney stone and Nigella sativa L. seeds protective effects**

Oxalate exposure up regulates the cyclooxygenase-2 (COX2) enzyme, the rate-limiting enzyme in prostaglandin synthesis (Jonassen et al., 2005 ▶), prostaglandins play a role in crystal binding and inflammation (Miyazawa et al., 2012 ▶). Furthermore, IĸBα, the endogenous inhibitor of NF-ĸB transcription factor is rapidly degraded by oxalate (Tozawa et al., 2008 ▶). Jonassen et al. ( 2005 ▶) showed that lipopolysaccharide exposure also induces similar response in renal tubular cells through activation of Toll-like receptor 4 (TLR4), while the matrix of many kidney stones contains endotoxin as the TLR4 ligand, even in non-infection stones. Moreover, it is noteworthy that TLR1-4 and TLR6 are also found on renal tubular epithelial cells (Anders et al., 2004). 

On the other hand, thymoquinone, an active quinone, that is the main component of *N. sativa* seeds, has antioxidant, free radical and superoxide anion scavenging and antibacterial effects, also it has anti-inflammatory effects, because it inhibits cyclooxygenase and 5- lipooxygenase pathways (Deepika et al., 2013 ▶). Moreover, the anti-diabetic (Fararh et al., 2002 ▶) anti-carcinogenic (Musa et al., 2015 ▶) and anti-ulcer (Jayakar, 2002 ▶) actions of thymoquinone have been shown in the literatures. Vance et al. (2008) ▶ showed that higher doses of thymoquinone were not as prooxidant and the *in vitro* treatment with thymoquinone increased the glutathione levels after 72 hr. However, the fixed oil of *N. sativa* has more powerful antioxidant and anti-eicosanoid properties than thymoquinone itself (Ali and Blunden, 2003 ▶).

Furthermore, Oberg et al. (2009) ▶ showed showed that a herbal melanin from *N. sativa* L. seed coats, modified cytokine production and was proposed as TLR4 ligand. The herbal melanin degraded IĸBα, induced caspase 8 cleavage and IL-8 and IL-6 production through NF-ĸB signaling in TLR4-transfected and TLR4-expressive cell lines. Plants and animals have melanin pigment which is found in seed coats, hair, inner ear, substantia nigra and fertilized ova. El-Obeid et al. (2006) ▶ reported that herbal melanin from *N. sativa* L. is a powerful stimulant of TLR4-expressing cells; therefore, it may have a potential role in infectious diseases and cancer (Liu et al., 2011 ▶). Melanin has antioxidative properties, for example in black fungi, *Aspergillus nidulans* or black sesame (Oncalves et al., 2005 ▶). Does the herbal melanin play a protective role in kidney stone disease? Answer to this question needs more investigation.

**Commercially available products**

A formulation of thymoquinone, cranberry extract and methionine has been patented in the USA to treat recurring cystitis or urologic syndrome. The most frequent form of the disease is called idiopathic lower urinary tract disease or idiopathic cystitis which is demonstrated as symptoms such as irritative voiding, hematuria or inappropriate micturition. Another type of urologic syndrome includes urolithiasis, urinary tract infection and less frequently, anatomic deformations or deficits (Pacioretty and Babish, 2011 ▶). 

Kidney diseases such as stones and renal failure and their consequences are progressively increasing in population. Despite new strategies in stone treatment, the recurrence rate is still high and renal failure has a severe impact on patients. Therefore, new interventions are necessary. Herbal medicine can be a useful approach to prevent the stone recurrence or improve renal failure. *N. sativa* L. is a promising species in this regard. The clinical trials about its effects on these renal situations are rarely documented and it is still required to test other components of the plant.

## Conflict of interest

There is no conflict of interests.
